# Scabies and risk of skin sores in remote Australian Aboriginal communities: A self-controlled case series study

**DOI:** 10.1371/journal.pntd.0006668

**Published:** 2018-07-25

**Authors:** Phyo Thu Zar Aung, Will Cuningham, Kerry Hwang, Ross M. Andrews, Jonathan R. Carapetis, Therese Kearns, Danielle Clucas, Jodie McVernon, Julie Ann Simpson, Steven Y.C. Tong, Patricia Therese Campbell

**Affiliations:** 1 Victorian Infectious Disease Reference Laboratory, The Royal Melbourne Hospital and The University of Melbourne, at the Peter Doherty Institute for Infection and Immunity, Melbourne, Victoria, Australia; 2 Menzies School of Health Research, Charles Darwin University, Darwin, Northern Territory, Australia; 3 Melbourne School of Population and Global Health, University of Melbourne, Melbourne, Victoria, Australia; 4 National Centre for Epidemiology & Population Health, Australian National University, Canberra, Australian Capital Territory, Australia; 5 Telethon Kids Institute, University of Western Australia and Perth Children’s Hospital, Perth, Western Australia, Australia; 6 Department of Paediatrics, The University of Melbourne, Melbourne, Victoria, Australia; 7 Murdoch Children’s Research Institute, The Royal Children’s Hospital, Melbourne, Victoria, Australia; 8 Victorian Infectious Diseases Service, The Royal Melbourne Hospital and The University of Melbourne, at the Peter Doherty Institute for Infection and Immunity, Melbourne, Victoria, Australia; University of California San Diego School of Medicine, UNITED STATES

## Abstract

**Background:**

Skin sores caused by *Group A streptococcus (GAS)* infection are a major public health problem in remote Aboriginal communities. Skin sores are often associated with scabies, which is evident in scabies intervention programs where a significant reduction of skin sores is seen after focusing solely on scabies control. Our study quantifies the strength of association between skin sores and scabies among Aboriginal children from the East Arnhem region in the Northern Territory.

**Methods and results:**

Pre-existing datasets from three published studies, which were conducted as part of the East Arnhem Healthy Skin Project (EAHSP), were analysed. Aboriginal children were followed from birth up to 4.5 years of age. Self-controlled case series design was used to determine the risks, within individuals, of developing skin sores when infected with scabies versus when there was no scabies infection. Participants were 11.9 times more likely to develop skin sores when infected with scabies compared with times when no scabies infection was evident (Incidence Rate Ratio (IRR) 11.9; 95% CI 10.3–13.7; p<0.001), and this was similar across the five Aboriginal communities. Children had lower risk of developing skin sores at age ≤1 year compared to at age >1 year (IRR 0.8; 95% CI 0.7–0.9).

**Conclusion:**

The association between scabies and skin sores is highly significant and indicates a causal relationship. The public health importance of scabies in northern Australia is underappreciated and a concerted approach is required to recognise and eliminate scabies as an important precursor of skin sores.

## Author summary

Skin sores, also known as impetigo, are highly contagious bacterial skin infections, which are found commonly in school children and occasionally in adults. Skin sores are prevalent in disadvantaged or resource-poor settings. In Australia, about two thirds of Aboriginal children suffer from skin sores by their first birthday. If untreated or treated poorly, skin sores can eventually cause heart and kidney problems. It is also believed that scabies, another common skin infection in Aboriginal children, can increase the risk of developing skin sores by allowing the bacteria to enter the skin more easily through breaks in the skin. Our research explored the following: if scabies is a risk factor for skin sores then what is the strength of the association between the two conditions.

## Introduction

Remote Aboriginal communities in northern Australia have the world’s highest prevalence of skin sores with more than 80% of children affected by their first birthday[1]. Skin sores, also known as impetigo, are commonly caused by Group A *Streptococcus* (GAS) infections in these populations and can have serious sequelae such as invasive bacterial infection and post-streptococcal glomerulonephritis, which in turn increases the risk of chronic renal disease [2].

Acquisition of skin sores is influenced by other skin infections, particularly scabies. Scabies is endemic in remote northern Australia and found in up to 35% of children and 25% of adults in the region [3]. Scabies infection often leads to a secondary GAS infection of the skin and scabies control is considered a priority in measures aimed at reducing skin sores in the Aboriginal population [4–7].

Kearns et al. found that skin sores were seven times more likely to be concurrently diagnosed with scabies than when there was no scabies diagnosis[1]. The risk ratio was calculated based on the diagnosis of skin sore infection at a presentation of scabies compared to no diagnosis of scabies at the same presentation. However, their study focused on the concurrent risk between scabies and skin sores and further research is needed to understand the temporal relationship between the two conditions.

Our study employed a self-controlled case series method to quantify the risk of scabies on skin sores using historical data from observational studies in Northern Territory. The study findings will contribute to the ongoing mathematical modelling research on understanding of GAS transmission and inform policy makers in prevention and control of skin sores and scabies in remote Aboriginal communities.

## Methods

### Data sources

We analysed pre-existing datasets from three published studies, which were conducted as part of the East Arnhem Healthy Skin Project (EAHSP) overseen by the Menzies School of Health Research [1, 8, 9]. The EAHSP was a regional collaboration to reduce skin infections among children in five remote Aboriginal communities in the East Arnhem region in the Northern Territory. The three studies, namely Kearns et al.[1], McMeniman et al.[9], and Clucas et al.[8], were separate but overlapping cohorts in the communities where EAHSP was conducted. These studies employed the same methodology to retrospectively review medical records from community health clinics in the region.

Despite their similar research methodology, the three studies varied in inclusion criteria and duration of follow-up (Table 1). In Kearns et al., eligible participants needed to have at least one clinic presentation in each quarter of their first year of life [1]. The duration of follow-up differed among the studies: participants were followed from birth to 1 year in Kearns et al., to 2-years in McMeniman et al., and up to 5-years of age in Clucas et al. One caveat of the Clucas et al. study is that although children born after 1 January 2001 were included, presentations were followed only from 1 January 2002, and therefore not all participants were followed from birth (28%) [8]. Complete descriptions of the research methods and study population for these studies are available elsewhere [1, 8, 9].

**Table 1:** Description of the datasets including follow-up duration and participant characteristics.

Study	Period for Clinic presentations	Follow-up duration	Number In dataset	Community				
				A	B	C	D	E
Kearns et al.	Jan-01 –Feb 07	Birth– 1 year of life	320	47	93	72	47	61
McMeniman et al.	Feb-01 –Jan 07	Birth– 2 years of life	99	-	-	99	-	-
Clucas et al.	Jan-02 –Sep 05	Birth– 4.75 years of Life^a^	174	77	-	-	-	97
Combined dataset	Jan-01 –Feb 07	Birth—4.75 years of life	417^b^	77	93	103	47	97

^a^Births were included from January 2001, but clinic records were only reviewed from January 2002 onwards i.e., not all participants were followed from birth (n = 48).

^b^The total number of children, i.e., 417, does not equate to the sum of the number of children in the three studies (Kearns, Clucas and McMeniman) as the study populations overlap with one another.

Data collected included date of presentation, child’s height and weight, any reason for presentation, antibiotic use, and referrals to hospital. Scabies were recorded as reasons for presentation if they were either noted specifically or with reference to scabies treatment given, while skin sores were recorded if there was any mention of skin sores or other presumed bacterial infections of the skin including boils, carbuncles, abscesses, ulcers and pustules (Clucas). Multiple presentations on the same day were recorded as a single event. We merged the datasets from three published studies into a single composite line-listed dataset. All data analysed were de-identified. Ethics approval was obtained from the Human Research Ethics Committee of the Northern Territory Department of Health and Families and the Menzies School of Health Research (Ethics approval 2015–2516).

### Self-controlled case series

Data on infection-free children i.e., who developed neither scabies nor skin sores were not available, in the three pooled studies, to determine the difference between the exposed and non-exposed groups using cohort methods. Therefore, we used self-controlled case series (SCCS) method as an alternative to cohort studies by comparing the risks during different time periods within individuals[10].

The SCCS method compares the risks of developing events in the periods following exposure versus non-exposed periods within individuals. This method controls for fixed (i.e. time-independent) confounders such as gender and ethnicity, and only requires cases for analysis [11, 12].

### Study population

Our study included children from five Aboriginal communities who attended the community health clinics from January 2001 to February 2007. We applied the following exclusions sequentially to our study cohort:

1. To improve data quality, we excluded individuals with less than 12 months of clinic data.
2. As clinic attendances were highest in their first year of life and to minimise selection bias, we excluded individuals if data on the first year of life were missing.
3. As only participants with events are required for self-controlled case series analysis, we excluded individuals with no diagnosis of skin sores at any time.
4. To preclude long intervals between clinic visits, we censored the observation period for each participant by setting the maximum duration between successive clinic visits as not more than 120 days.
5. Records were re-reviewed following censoring to identify any children whose infection episodes had been removed, leaving them without a diagnosis of skin sores during the observation period (as in step 3).

### Exposure and outcome

The observation period ran from birth to the earliest of (i) the first time the observation period went for more than 120 days between clinic visits; (ii) the end of the study follow-up period; or (iii) the maximum age for their respective study (Table 1). The observation period was further divided in two age groups: ≤1 year and >1 year (up to 4 years old) to account the effect of age on both exposure and outcome. Scabies and skin sores episodes were identified from the clinic records. We assessed, within individuals, the risk of developing skin sores following scabies infection (exposed period) versus the risk of skin sores infection when there is no scabies (non-exposed period).

**Fig 1:**
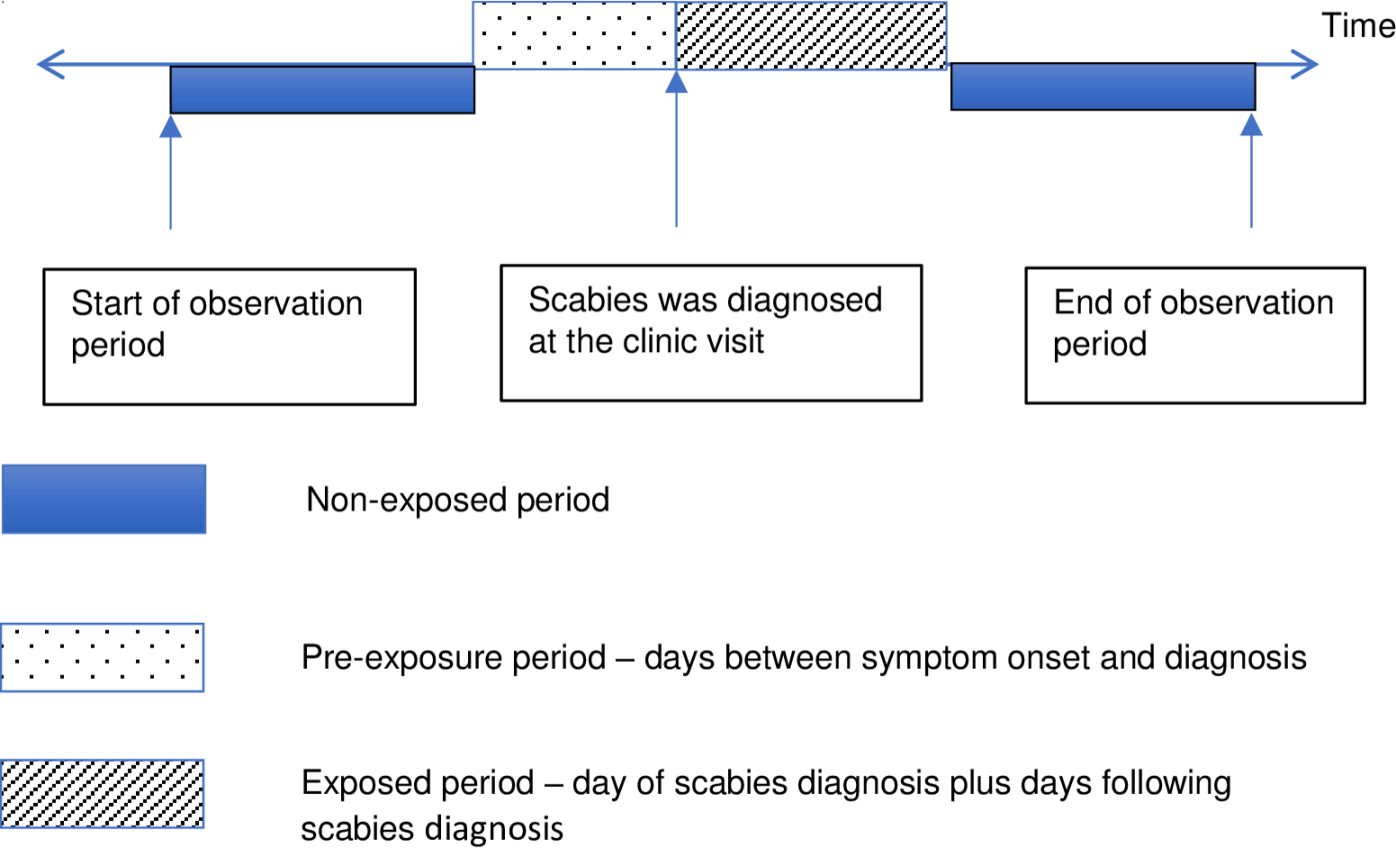
Self-controlled case series (SCCS) model with observation period, baseline period and risk periods.

The period with the risk of developing skin sores was further subdivided into two segments: pre-exposure period and exposed period. The pre-exposure period is the period before the diagnosis of scabies (the time taken from onset of scabies symptoms to the diagnosis of scabies by the clinician) while the exposed period is the day of the diagnosis of scabies plus a specified period following the scabies diagnosis (Fig 1). The pre-exposure period is included to account for the presence of scabies prior to clinic attendance and any delay in the diagnosis of scabies.

Our baseline assumption was that the pre-exposure period was seven days and exposed period was 14 days. These periods were defined based on the first diagnosis of scabies and any subsequent episodes that fell within the same risk period, were counted as one infection. Skin sores episodes were then mapped in relation to different time periods. A seven-day duration was chosen for the pre-exposure period based on the shortest time period reported in the literature [13, 14] and the very frequent presentation of our participants for medical attendance (on average every two weeks) [1, 8, 9].

The natural history of skin sores is that an uncomplicated impetigo heals spontaneously within two weeks [15, 16]. Detailed studies of the natural history of impetigo primarily caused by S. pyogenes found that the mean time to spontaneous healing was 12.6 days, with a range of 6–31 days [17]. These data and assumptions may not be generalisable to impetigo in non-endemic settings where *Staphylococcus aureus* is the primary pathogen. In our study, we assumed that repeat clinic presentations, positive for skin sores within a two-week period, were the same infectious episode and only the first episode was included in the analysis.

### Data analysis

We transformed the data into a time series format and applied deterministic imputation to substitute missing data as follows. The imputation was performed in a stepwise approach. Firstly, we found the first event (skin sores or scabies) in an individual and carried the value forward for the following 14 days. This step was repeated for any recurring events within the same individual. After that, any missing data were assumed as non-events. This was to ensure that skin sores and scabies events within 14 days were counted as one episode.

Our primary analysis involved estimating the relative incidence rate of skin sores during exposed periods compared to the non-exposed period. Conditional Poisson regression was used to calculate incidence rate ratios (IRRs) and 95% confidence intervals (CIs). Analyses were undertaken using Stata/IC version 14 (StataCorp. College Station, TX, US). We converted the time periods from years to days for higher granularity and better accuracy. We undertook sensitivity analyses by increasing the exposed period from 14 to 21 and 28 days while keeping the pre-exposure interval consistent. We further analysed the incidence rate ratios within each community.

## Results

Among the initial study population of 417 children, 55.9% (n = 233) were male. The number of clinic attendances ranged from 1 to 117 with median attendance of 19 times per individual during the six-year follow-up period from January 2001 to February 2007. Approximately 75% of children had their first episode of skin sores before their first birthday.

**Fig 2:**
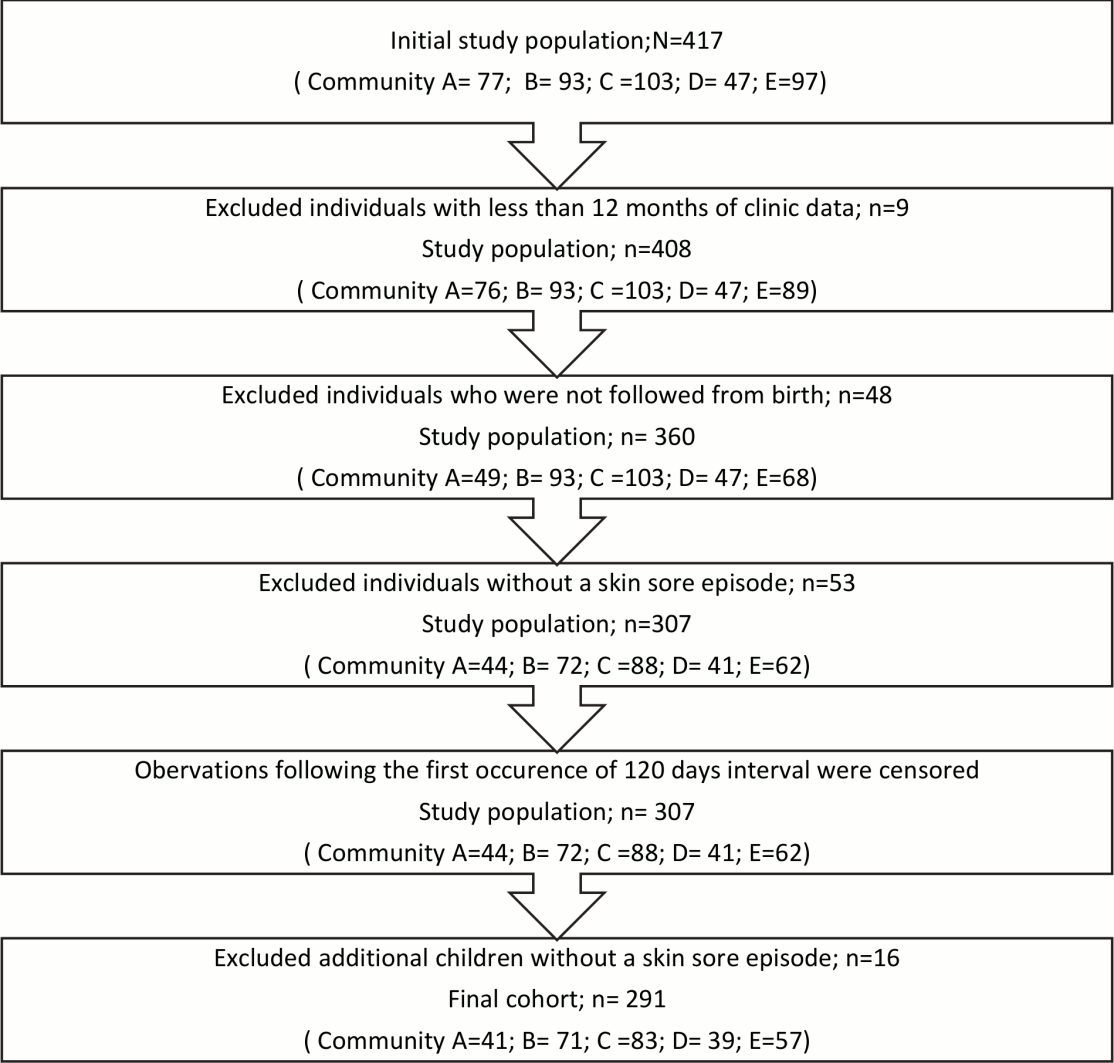
Identification of study participants eligible for Self-controlled case series (SCCS) analysis.

After a series of exclusions, we arrived at a study population of 307 children (Fig 2). Censoring the observation period for each child once they went more than 120 days between clinic visits dropped skin sores episodes beyond the censored date, resulting in more children without a skin sore episode. Therefore, an additional 16 children were excluded resulting in a total of 291 children in the final analysis (Fig 2).

### Relative incidence of skin sores

Compared to the non-exposed periods, the overall rate of skin sores was increased in both pre-exposure and exposed periods, with a significant increase in the exposed periods. Although the pre-exposure periods were associated with a higher prevalence of skin sores than baseline non-exposed periods (IRR 1.3; 95% CI 0.8–1.9), this finding was not statistically significant (p = 0.296). However, children were 11.9 times more likely to develop skin sores during the exposed periods compared with the baseline non-exposed periods (IRR 11.9; 95% CI 10.3–13.7; p<0.001) (Table 2). When exposed to scabies, children had lower risk of developing skin sores at age ≤1 year compared to at age >1 year (IRR 0.8; 95% CI 0.7–0.9).

**Table 2:** Sensitivity analysis for the incidence rate ratios (IRR) of skin sores in pre-exposure periods and exposed periods.

	Duration of exposed period		
	14 days	21 days	28 days
	IRR (95%CI; p value)	IRR (95%CI; p value)	IRR (95%CI; p value)
**Risk periods**			
Pre-exposure period (Fixed period of 7 days)	1.3(0.8–1.9; 0.30)	1.2(0.7–1.8; 0.54)	1.2(0.8–1.9; 0.39)
Exposed period	11.9(10.3–13.7; 0.00)	8.8(7.6–10.1; 0.00)	7.6 (6.6–8.8; 0.00)
**Age groups** (>1 year as baseline)			
0–1 year	0.8(0.7–0.9; 0.00)	0.8(0.7–0.9; 0.00)	0.8(0.7–0.9: 0.00)

CI–Confidence Interval

Sensitivity analysis on the duration of the exposure period (14 days, 21 days and 28 days) showed that increasing the length of the exposed period resulted in a progressive decrease in the incidence rate ratio for skin sores associated with scabies, compared with the baseline (Table 2). However, the study outcomes for pre-exposure periods and age groups did not change significantly (Table 2). Community stratified rates in five communities (using the primary analysis assumption of 14-day exposure period) showed similar results to the overall rates in both pre-exposure and exposed periods, with overlapping confidence intervals (Table 3).

**Table 3:** Incidence rate ratios (IRR) of skin sores in pre-exposure periods and exposed periods, among five Aboriginal communities.

	Community				
	A (n = 77)	B (n = 93)	C (n = 103)	D (n = 47)	E (n = 97)
	IRR (95%CI; p-value)	IRR^a^ (95%CI; p-value)	IRR (95%CI; p-value)	IRR (95%CI; p-value)	IRR (95%CI; p-value)
**Risk periods**					
Pre-exposure period	2.5(0.96.9; 0.09)	1.4(0.4–4.6; 0.56)	1.4(0.6–3.3; 0.39)	1.1(0.3–3.8; 0.82)	0.6(0.2–1.9; 0.39)
Exposed period	8.6(5.8–12.6; 0.00)	9.7(6.7–14.1; 0.00)	8.8(6.8–11.3; 0.00)	8.2(5.3–12.6; 0.00)	5.1(3.9–6.7; 0.00)
**Age group** (>1 year as baseline)					
0–1 year	0.6(0.4–0.9; 0.2)	-	0.9(0.8–1.3; 0.87)	-	0.7(0.5–0.9; 0.01)

CI—Confidence Interval

^a^Not adjusted for age group because the data from these communities, participating in the study by Kearns et al., only included children in the first 12 months of life

## Discussion

Our study is the first to use the self-controlled case series method to investigate the association between scabies and skin sores. We found that, when infected with scabies, children were 12 times more likely to develop skin sores than in the absence of scabies infestation. Our findings reiterate the extreme burden of skin disease in remote Aboriginal and Torres Strait Islander communities, with up to 75% of children in these communities having a skin sore by their first birthday. This force of infection is compatible with data from low and middle-income regions such as India and those in the South Pacific where the prevalence of skin sores among preschool children and adolescents ranges from 42% to 70% [13].

The risk estimate from our study is substantially higher than previously reported relative risks of scabies and skin sores co-infections, which ranged from 2.4 to 7.0 [1, 3, 13, 18]. There are two possible explanations. Firstly, earlier studies analysed risk based on concurrent presentation with scabies and skin sores—our study extended the hypothesised association to a temporal relationship between diagnosed scabies and skin sores, within a defined risk window. Secondly, the SCCS method has the advantage of controlling implicitly for fixed confounders, which can affect case-control and cohort studies, and would be anticipated to report a more faithful estimate of true risk [19].

Whilst our study cannot establish causality definitively, it has a number of attributes supporting a causal relationship between scabies and skin sores. These include the significant association and the temporality between the two conditions and consistency with earlier studies. These results are complemented by the sensitivity analyses on the duration of the post diagnosis scabies exposure period. We found that lengthening the exposure window from 14 to 21 days and then to 28 days was associated with a decline in the incidence rate ratio. This strengthens inference on the purported causal relationship between scabies and skin sores, i.e., if scabies truly increases the risk of skin sores, it is logical that the risk of this outcome will occur within a time frame more narrowly associated with the diagnosis (i.e. 14 days rather than 28 days).

One of the strengths of our study is inclusion of pre-exposure period in the analysis so as to reduce the inflation of relative risks in the exposed period. We observed that the risk of developing skin sores in pre-exposure periods is much lower than that in exposed periods. However, the results for pre-exposure periods are not statistically significant, and furthermore, these findings are probably due to clinic nonattendance rather than actual absence of infections.

We found that children were less likely to develop skin sores in their infanthood than when they were between one and four years of age. This is consistent across different durations of scabies infection. One possibility is that infants are less likely to scratch and develop excoriation of the skin when infected with scabies than older children, thereby reducing the incidence of skin sores. However, it is important to note that the overall association between skin sores and scabies remains significant regardless of age.

Our finding is of a link at the individual level between scabies and skin sore risk. Studies of skin sores in the East Arnhem region have identified GAS as the dominant pathogen in around 80% of cases [2], and clearance of GAS has been identified as the only independent predictor of complete resolution of skin sores [20]. It is therefore likely that the observed association between scabies and skin sores reflects a heightened risk of streptococcal infection in the presence of scabies, but we can neither confirm nor refute a similar association with staphylococcal infection on the basis of the evidence here presented. The findings are unlikely to be generalisable to non-endemic settings for impetigo where *S. aureus* is usually the primary pathogen.

Our study limitations include that, in the SCCS design, occurrence of an event should not affect subsequent exposures. Although research is limited in the area of scabies and skin sores, to date, there is no evidence suggesting the impact of skin sores on the risk of developing scabies. Therefore, we assumed that a skin sore infection was unlikely to affect subsequent scabies exposure. However, our exposures and events are defined by diagnosed cases and may be influenced by clinic attendances. For example, if a diagnosis of skin sores prompted a participant to return to the clinic for clearance check-up, these follow-up visits may increase the chance of scabies being detected incidentally.

Secondly, our study has likely underestimated the incidence of scabies as we only included the children who presented to the clinics. Furthermore, we did not control for any potential time varying confounders, which can cause delay in diagnosis such as stigma associated with scabies, restricted access to health care and chronic steroid use causing masked presentation[21–23].

Thirdly, our study relies on the documentation and clinical diagnosis of the clinicians. While there is imprecision involved in relying on syndromic reporting of skin sores and clinically observed scabies identified from an historical clinic record review, this level of diagnostic uncertainty reflects the reality of clinical practice in a remote setting. We anticipate that the high prevalence of both conditions in the communities studied should increase clinical experience and the consistency and reliability of observer reporting. Indeed, the involved communities and associated healthcare clinics have had a long standing interest in skin health related research, and thus have well engaged clinicians around the diagnosis and management of impetigo and scabies. Moreover, our use of the self-controlled case series method accounts for any variability between participants.

Lastly, whilst the SCCS method provides a robust study design, we may not be able to generalise the findings conclusively. Our findings are based on a specific subgroup of Aboriginal children and may not be representative of the wider Australian population or older age groups nor perhaps of Aboriginal children living in other remote communities. Furthermore, SCCS method was first developed for rare events and historically used in vaccine trials, yet we assessed two common conditions in Aboriginal communities. However, others have utilised the SCCS method for common events such as drug safety and have produced findings consistent with those of previous studies, which suggests the SCCS method was appropriate for our study[24, 25].

The self-controlled case series method we have used is potentially transferrable to other settings where data have been collected that do not invalidate the necessary conditions. By investigating associations within an individual, the self-controlled case series method implicitly controls for contextual factors that may influence the risk of either infection, making findings more comparable across situations and settings. As regards to public health implications, our findings robustly support existing inference on the contribution of scabies to skin sore risk, particularly in the very young [13, 14]. Accordingly, we endorse holistic healthy skin strategies that raise awareness of the need for prevention, early presentation and multi-pronged treatment strategies to reduce the overall burden and the long-term sequelae of skin disease.

In conclusion, our study demonstrated that the association between scabies and skin sores is significant, more so than previously reported in standard cohort and case- controlled studies. A concerted approach is needed in implementing scabies elimination programs to prevent skin sores, particularly due to GAS, and their devastating complications in remote Aboriginal communities.

## Supporting information

S1 Checklist STROBE checklist. (DOC)

S1 Dataset (XLS)
