# Supplementary material for: Scabies and risk of skin sores in remote Australian Aboriginal communities: A self-controlled case series study
Source: PLoS Negl Trop Dis. 2018 Jul 25;12(7):e0006668. doi: 10.1371/journal.pntd.0006668 (PMC6078322; doi:10.1371/journal.pntd.0006668)
Supplement: S1 Checklist — (DOC) [file pntd.0006668.s001.doc]

STROBE Statement—Checklist of items that should be included in reports of ***cohort studies***

|  | Item No | Recommendation | Reported on |
| --- | --- | --- | --- |
| **Title and abstract** | 1 | (*a*) Indicate the study’s design with a commonly used term in the title or the abstract | Title, paragraph 1, page 1 |
|  |  | (*b*) Provide in the abstract an informative and balanced summary of what was done and what was found | Abstract, paragraph 2, page 3 |
| Introduction | | |  |
| Background/rationale | 2 | Explain the scientific background and rationale for the investigation being reported | Introduction, paragraph 1-3, page 5 |
| Objectives | 3 | State specific objectives, including any prespecified hypotheses | Introduction, paragraph 3-4, page 5 |
| Methods | | |  |
| Study design | 4 | Present key elements of study design early in the paper | Self-controlled case series, paragraph 1-2, page 7 |
| Setting | 5 | Describe the setting, locations, and relevant dates, including periods of recruitment, exposure, follow-up, and data collection | Data sources, paragraph 1-3, page 6 |
| Participants | 6 | (*a*) Give the eligibility criteria, and the sources and methods of selection of participants. Describe methods of follow-up | Data sources, paragraph 2, page 6 |
|  |  | (*b*) For matched studies, give matching criteria and number of exposed and unexposed | Not applicable |
| Variables | 7 | Clearly define all outcomes, exposures, predictors, potential confounders, and effect modifiers. Give diagnostic criteria, if applicable | Methods, paragraph 3, page 6; paragraph 2, page 7; paragraph 1, page 8 |
| Data sources/ measurement | 8* | For each variable of interest, give sources of data and details of methods of assessment (measurement). Describe comparability of assessment methods if there is more than one group | Data sources, paragraph 1-3, page 6; Table 1, page 9 |
| Bias | 9 | Describe any efforts to address potential sources of bias | Study population, paragraph 3, page 7 |
| Study size | 10 | Explain how the study size was arrived at | Study population, paragraph 3, page 7 |
| Quantitative variables | 11 | Explain how quantitative variables were handled in the analyses. If applicable, describe which groupings were chosen and why | Exposure and outcome, paragraph 1, page 8; paragraph 1, page 9. |
| Statistical methods | 12 | (*a*) Describe all statistical methods, including those used to control for confounding | Data analysis, paragraph 3-4, page 10-11 |
|  |  | (*b*) Describe any methods used to examine subgroups and interactions | Data analysis, paragraph 4, page 10-11 |
|  |  | (*c*) Explain how missing data were addressed | Data analysis, paragraph 3, page 10 |
|  |  | (*d*) If applicable, explain how loss to follow-up was addressed | Not applicable |
|  |  | (*e*) Describe any sensitivity analyses | Data analysis, paragraph 4, page 10-11 |
| Results | | |  |
| Participants | 13* | (a) Report numbers of individuals at each stage of study—eg numbers potentially eligible, examined for eligibility, confirmed eligible, included in the study, completing follow-up, and analysed | Results, figure 2, page 11 |
|  |  | (b) Give reasons for non-participation at each stage | Results, figure 2, page 11 |
|  |  | (c) Consider use of a flow diagram | Results, figure 2, page 11 |
| Descriptive data | 14* | (a) Give characteristics of study participants (eg demographic, clinical, social) and information on exposures and potential confounders | Results, paragraph 1, page 11 |
|  |  | (b) Indicate number of participants with missing data for each variable of interest | Results, figure 2, page 11 |
|  |  | (c) Summarise follow-up time (eg, average and total amount) | Results, paragraph 1, page 11 |
| Outcome data | 15* | Report numbers of outcome events or summary measures over time | Relative incidence of skin sores, paragraph 3, page 11 |
| Main results | 16 | (*a*) Give unadjusted estimates and, if applicable, confounder-adjusted estimates and their precision (eg, 95% confidence interval). Make clear which confounders were adjusted for and why they were included | Results, table 2, page 12 |
|  |  | (*b*) Report category boundaries when continuous variables were categorized | Relative incidence of skin sores, paragraph 3, page 11-12 |
|  |  | (*c*) If relevant, consider translating estimates of relative risk into absolute risk for a meaningful time period | Not applicable |
| Other analyses | 17 | Report other analyses done—eg analyses of subgroups and interactions, and sensitivity analyses | Results, paragraph 1, page 12; table 3, page 13 |
| Discussion | | |  |
| Key results | 18 | Summarise key results with reference to study objectives | Discussion, paragraph 1, page 13 |
| Limitations | 19 | Discuss limitations of the study, taking into account sources of potential bias or imprecision. Discuss both direction and magnitude of any potential bias | Discussion, paragraph 2-4, page 15 |
| Interpretation | 20 | Give a cautious overall interpretation of results considering objectives, limitations, multiplicity of analyses, results from similar studies, and other relevant evidence | Discussion, paragraph 1-3, page 14; paragraph 1-4, page 15 |
| Generalisability | 21 | Discuss the generalisability (external validity) of the study results | Discussion, paragraph 4, page 15 |
| Other information | | |  |
| Funding | 22 | Give the source of funding and the role of the funders for the present study and, if applicable, for the original study on which the present article is based | Online submission form, financial disclosure |

*Give information separately for exposed and unexposed groups.

**Note:** An Explanation and Elaboration article discusses each checklist item and gives methodological background and published examples of transparent reporting. The STROBE checklist is best used in conjunction with this article (freely available on the Web sites of PLoS Medicine at http://www.plosmedicine.org/, Annals of Internal Medicine at http://www.annals.org/, and Epidemiology at http://www.epidem.com/). Information on the STROBE Initiative is available at http://www.strobe-statement.org.
